# "Sleeping Sickness" in Uganda

**Published:** 1902-12-27

**Authors:** 


### The Hospital.

A Journal of

The Medical Sciences and Hospital Administration.

Vol. XXXIII.—No. 848.] Edited by Sir Henry Burdett, K.C.B., and Solomon C. Smith, M.D., M.R.C.P. [December 27, 1902.

### "Sleeping Sickness" in Uganda.

It has been said by some cynical individual that the average inhabitant of these islands would feel the loss of his own little finger more acutely than he would the intelligence that the whole of China had been destroyed by some convulsion of Nature. There is sufficient truth in the observation to illustrate that it is, indeed, somewhat difficult to realise the nature and extent of sufferings from which we are separated by great distances, and which befall those who are comparatively unknown to us, but, even when full allowance is made for such circumstances as these, the conditions known to be existing in Uganda are such as should call forth not only the sympathy, but the active assistance of the English nation, under whose Protectorate the government of the great country concerned is now conducted. The recent appearance and rapid extension there of the disease popularly known as "sleeping sickness," or as "negro lethargy," has induced the Foreign Office to send out a Commission, selected by the Royal Society, and under the direction of Dr. Low, Cragg's research scholar of the London School of Tropical Medicine, to investigate the conditions in which this extension has occurred; and Dr. Low, who has just returned to England, leaving the bacteriologist of the Commission to pursue further inquiries, brings back with him a narrative for which it would be hard to find parallels even among the histories of the epidemics of the Middle Ages. He estimates that the disease, which first made its appearance in the Protectorate a "few years" ago, has since that time destroyed the lives of from twenty to thirty thousand people; and he tells us that it has produced a depopulation under which great tracts of land have passed out of cultivation, and which already seriously affects the financial resources of the country.

It requires some imagination to realise, here in England, that the little coloured patch marked as "Uganda" in the maps, and lying along the north-west shore of the Victoria Nyanza, is a State containing a population of about five millions, and sufficiently favoured by nature to have been called by Stanley "the Pearl of Africa." The country presents agreeable diversities of mountain, forest, and plain, is well wooded and very fertile, and has a singularly mild and uniform climate, the temperature ranging from 50° to 90° during the year; the railway from Mombasa is making rapid strides into the district, and the conditions hold out great promise of a future of active commerce which is now seriously threatened by the progress of the epidemic.

The so-called "sleeping sickness" has been known for many years as an endemic disease of the West Coast of Africa, usually confined to the belt lying between Senegambia on the north of the equator, and Loanda on the south, but extending into the Congo at least as far as to Stanley Falls; and it was formerly occasionally imported into the West Indies, but has not been seen there since the abolition of the slave trade. Between Uganda and the Congo there is no trade route, and the precise time and manner of the introduction into Uganda are unknown. The attendant phenomena have been seized upon by one or two writers of fiction, but, until lately, have hardly been made the subjects of serious scientific inquiry. The results of such inquiry appear to show that the malady is essentially a chronic form of meningitis, that it is produced by the presence of a "germ" presumably bacterial, and that it is communicable from person to person through channels which have not as yet been clearly demonstrated, the general fact of infectiveness not being doubtful. It seems to differ widely from the only form of epidemic meningitis known in Europe, the so-called "cerebro-spinal," in its chronic character, as well as in its almost invariably fatal issue, recovery or cure being practically unknown. The early symptoms are so slight that they would easily escape the notice of an unpractised observer; but the natives of Uganda have become thoroughly acquainted with them, and have brought to the hospital of the Commission many commencing cases in which their diagnosis has been only too surely justified by the event. The duration varies from one month to six, and the disease is described by Dr. Low as being capable of being classed with hydrophobia as one of the most fatal that is known to mankind. More or less slowly, the early listlessness passes into coma, and the coma into death, with occasional deceptive periods of apparent improvement. No treatment appears to exert any definite influence upon either the march or the termination of the malady. A precise statement of the results obtained by the Commission will, we understand, be laid before the Royal Society as soon as possible, and prior to publication elsewhere; but, in the meantime, the Commissioner of the State, Colonel Sadler, is doing whatever is possible to check the advance of the disease by the enforcement of isolation; the great fear being that it may ultimately reach the railway, and may thus be carried out of the country by the way of Mombasa. So far, the only cases observed have been in people of negro race; but there can be no confidence that Europeans would be exempt if they were continuously exposed to the presumed infection.

